# The Baboon (*Papio spp*.) as a Model of Human Ebola Virus Infection 

**DOI:** 10.3390/v4102400

**Published:** 2012-10-23

**Authors:** Donna L. Perry, Laura Bollinger, Gary L.White

**Affiliations:** 1 Integrated Research Facility, Division of Clinical Research, NIAID, NIH, Frederick, MD, USA; Email: bollingerl@niaid.nih.gov; 2 Department of Pathology, University of Oklahoma Baboon Research Resource, University of Oklahoma, Ft. Reno Science Park, OK, USA; Email: gary-white@ouhsc.edu

**Keywords:** *Filoviridae*, Filovirus, Baboon, Papio spp., Ebola virus, Marburg virus, BSL-4

## Abstract

Baboons are susceptible to natural Ebola virus (EBOV) infection and share 96% genetic homology with humans. Despite these characteristics, baboons have rarely been utilized as experimental models of human EBOV infection to evaluate the efficacy of prophylactics and therapeutics in the United States. This review will summarize what is known about the pathogenesis of EBOV infection in baboons compared to EBOV infection in humans and other Old World nonhuman primates. In addition, we will discuss how closely the baboon model recapitulates human EBOV infection. We will also review some of the housing requirements and behavioral attributes of baboons compared to other Old World nonhuman primates. Due to the lack of data available on the pathogenesis of Marburg virus (MARV) infection in baboons, discussion of the pathogenesis of MARV infection in baboons will be limited.

## 1. Introduction

Baboons and macaques are in the family *Cercopithecidae* (Old World monkeys), subfamily *Cercopithecinae*. Five subspecies of baboons range throughout subSaharan Africa and include: *Papio hamadryas anubis*, *Papio hamadryas cynocephalus*, *Papio hamadryas hamadryas*, *Papio hamadryas papio*,and *Papio hamadryas ursinus*. *Papio hamadryas anubis* is the most common subspecies used in research in the United States followed by *Papio hamadryas cynocephalus.*

The baboon possesses a number morphologic and physiologic characteristics that closely resemble human beings and, as a result, they are one of the most commonly used nonhuman primates (NHPs) for xenotransplant research [1,2,3,4]. Adult male baboons often exceed 25 kg and adult females range in weigh from 12 to 18 kg, making them appreciably larger in size than macaques and other Old World monkeys. Their size makes them particularly well-suited for a number of clinical procedures, including endoscopy and surgery. The menstrual cycles of baboons are continuous, and their prominent sex skin directly correlates with the stage of the estrus cycle, facilitating timed breeding [5]. Their placentation is identical to that of humans (discoid, hemochorial), making them especially useful for research involving various aspects of maternal-fetal transfer, including *in utero* vaccination and fetal blood sampling [6,7]. 

Although the baboon might appear to be more aggressive because of their larger size and large canine teeth, most investigators consider the baboons to be easier to work with in the laboratory setting than macaques [8]. The husbandry and housing requirements for baboons are similar to those of macaques. Cage sizes required for baboons depend on their age and size. Adult males and females are typically housed in Group 5 or 6 primate cages. Many biomedical research studies utilize baboons 1–3 years of age weighing 3–12 kg. These baboons require a smaller cage (Group 4) when singly housed. Baboons are typically fed an Old World monkey commercial diet used for macaques, and the diet is supplemented with fruits and vegetables. Cost of baboons varies with size and age but they are similar in cost to Indian-origin rhesus macaques (approximately $4000–7000) and more expensive than cynomolgus macaques (*Macaca fascicularis*). Domestically bred baboons (*Papio hamadryas* anubis) are available in the United States in limited quantities from two programs supported by the National Institutes of Health (NIH). One of the NIH supported colonies has a very limited number of specific pathogen-free baboons (negative for thirteen endogenous viruses including all five known herpes viruses, four retroviruses, four other baboon viruses, and the most common baboon parasites) [9]. 

The great apes are most closely related to man, with an estimated 1.3% DNA sequence difference [10] while baboons have an approximately 4% DNA sequence difference [11]. Baboons are genetically closer to man than other Old World monkeys, such as macaques which have a 6.46% DNA sequence difference [10]. In addition, baboons produce all four isotypes of IgG antibody (IgG1-4). The opsonizing IgG3 antibody isotype is not expressed in macaques [12,13]. This IgG3 isotype is thought to be essential in the immune response against protein antigens and is integral in the control and recovery from some viral infections [12,14]. 

Baboons have been utilized for vaccine development against viruses such as human immunodeficiency virus 1, respiratory syncytial virus, West Nile virus, Rift Valley fever virus, and hepatitis C virus [11,15,16]. Baboons have also been used as a model for human sepsis for over 40 years because the clinicopathologic changes that occur in septic baboons closely resemble those seen in man [17,18,19,20,21]. The baboon sepsis model has led to approved therapeutic agents now used in clinical practice [8]. 

Ebola virus seropositive (IgG antibodies) baboons are found in the wild [22], but despite their susceptibility to natural infection, their use as a model of human infection in the United States has been limited. Using the few baboon EBOV studies (largely in the former USSR) [23,24,25,26], we will compare and contrast what is currently known about EBOV infection in baboons with human EBOV infections and address some of the reasons that underlie the scarce utilization of baboons in filovirus research. 

## 2. Filovirus Hemorrhagic Fevers

The family *Filoviridae* includes two genera, *Marburgvirus* (marburgviruses) and *Ebolavirus* (ebolaviruses). “*Cuevavirus*” was recently suggested as a third genus. Two marburgviruses, Marburg virus (MARV) and Ravn virus (RAVV), and four ebolaviruses, Bundibugyo virus (BDBV), Ebola virus (EBOV), formerly the Zaire strain, Sudan virus (SUDV), and Taï Forest virus (TAFV), are known to cause viral hemorrhagic fever in humans [27,28]. The most common and the best characterized human infections are those caused by MARV, EBOV, BDBV, and SUDV. Although MARV and EBOV share less than 50% genetic homology, the symptoms associated with early infection and the hemorrhagic diatheses that sometimes occur during the disease course are clinically indistinguishable [27]. These symptoms, however, are not pathognomonic for infection with either virus, as syndromes caused by nonsegmented, enveloped, single-stranded RNA viruses in the *Arenaviridae*, *Bunyaviridae,* and *Flaviviridae* families share these clinical features and, as a result, are also considered “viral hemorrhagic fevers” [29,30,31]. 

### 2.1. Clinical Signs

Ebolavirus infection occurs through direct contact, contact with fomites via breaks in the skin, or through exposure to aerosolized virus. Venereal transmission is also a suspected, but not a proven, mode of transmission. Filovirus can be isolated from the urine and semen, in addition to blood and oral secretions, even during an extended convalescent period [32,33]. Following infection, the incubation period is 2 to 21 days during which virus replication and systemic dissemination occur via circulating monocytes, tissue macrophages, and dentritic cells [34]. Expression of cell surface tissue factor and release of a variety of cytokines and chemokines (e.g., tumor necrosis factor α, eotaxin, macrophage inflammatory protein-1α, interleukin [IL]-1β, IL-6, IL-10, IL-15, IL-16, IL-1 receptor antagonist), reactive oxygen species, and nitric oxide are stimulated in infected cells resulting initially in flu-like symptoms: fever (39–40 °C), myalgia, fatigue, headache, and sore throat [35]. A peripheral lymphadenopathy involving the submandibular, axillary, cervical, and nuchal lymph nodes is observed in some patients [32]. In 25–50% of afflicted humans, a rash typically develops focally 5 to 8 days after the initial flu-like symptoms and progresses to a generalized rash; its presence seemingly coincident with systemic viral spread. This rash has been variably described as maculopapular, morbilliform, and scarletinoid [32,36,37,38,39,40,41,42,43]. 

Later in the disease course, anorexia, abdominal pain, emesis, and diarrhea occur, followed by conjunctivitis, jaundice, and sometimes unexplained hemorrhages. Although filoviruses are known for the bleeding diatheses that sometimes occur following infection, epistaxis, hematemesis, and hematochezia are reported in less than 10–20% of patients [33,39,42,44,45,46,47]. The widespread, severe hemorrhage seen in some patients appears clinically as dermal petechiae and ecchymoses, hyposphagma, epistaxis, hematemesis, melena, vaginal bleeding, and excessive hemorrhage from venipuncture sites [36,38,48,49]. The virus infects multiple organs including the gastrointestinal tract, heart, liver, adrenal glands, kidneys, and nervous system. Some patients exhibit central and peripheral nervous system signs such as paresthesia, agitation, delirium, disinhibition, or convulsions [32]. These clinical signs may be the result of intracerebral or intracranial hemorrhage or viral encephalitis. It is currently not known if filoviruses infect neurons or supporting glial cells [50]. By end of the first week of infection, most patients show a progressive decline in urine output commonly resulting terminal oliguria [45,47,48,51]. Pregnant women abort [50,52]; however, a pathologic assessment of the uterus, placenta, and abortus has not been described. Therefore, it is not known if a direct virus-associated metritis, placentitis, or transplacental filovirus infection of the fetus occurs. 

### 2.2. Clinical Pathology

At initial presentation a granulocytosis with lymphopenia has been described in filovirus infected patients that progresses to a left shift [37,48,53,54,55]. Lymphocytes are not infected by the virus, and the cause of the lymphopenia is not known. However, this lymphopenia is thought to impede the adaptive immune response, and the mechanism may be apoptotic or pyroptotic lympholysis rather than being secondary to virus-induced cytopathic effects [49,55]. As the disease progresses, hyposegmented neutrophils (pseudo-Pelger-Huët cells) and atypical circulating lymphocytes and plasma cells that appear lymphoblast-like and plasmacytoid have been described [41,55,56].

The manifestation of bleeding diatheses secondary to filovirus infections is a characteristic, but not a consistent, finding in humans; such diatheses are associated with a poorer prognosis [32,39,42,45,54]. Coagulation abnormalities begin with the widespread release of tissue factor and cytokines from infected cells of the monocyte/macrophage system that increase vascular permeability and promote fibrin deposition from disease onset [57]. Fibrin deposition in combination with thrombocytopenia and the production of fibrin degradation products lead to a hypercoagulable state that culminates in disseminated intravascular coagulation (Table 1). Interestingly, endothelial cells do not appear to be primary targets of filovirus infection, and if infection of endothelial cells occurs at all, it is thought to occur late during the disease course [35,49,58]. Prolonged prothombin and activated partial thromboplastin times and elevated concentrations of fibrin degradation products have been described in patients infected with MARV and EBOV [36,38,48,49]. The development of thrombocytopenia in patients with filovirus infection is variable and may occur early or late in the disease course and sometimes persists until death [48,56,59]. However, in patients with TAFV, platelets counts have rebounded during recovery [60].

**Table 1:** Coagulation Parameters in Humans with Filovirus Infections.

Virus Country of origin	Marburg Uganda [56]	Marburg Uganda [61]	Marburg Rhodesia [59]	Marburg Uganda [54]	EbolaZaire [62]	Taï Forest Côte d'Ivoire [60]	Ebola Gabon [48]	Sudan Uganda [51]	EbolaZaire [43]
**# patients infected**	2 (gender NA)	1 M doctor	1M	1W	1 W	1 W	1 W	112 (gender NA)	2 (gender NA)
**Patient outcome**	Died (d 8-16)	Discharged d 32	Died d 7	Died d 9	Died d 10	Discharged d 15	Died d 23	55 F ^a^	2 died (d 8)
								57 NF ^b^	
**Bleeding time (min)** **Normal: <7 min [63] **									
**• hospital admission**		2 (d 5 at home)			7.5 (d 5)				
**• late infection**					10 (d 7) w/vitamin K				
**Coagulation time (min)** **Normal: <10 min [64]**									
**• hospital admission**		6.5 (d 5 at home)			9 (d 5)				
**• late infection**					12 (d 7) w/vitamin K				
**aPTT (s)^c^** **normal: 29** **–** **40 s ^d^**						Normal throughout			
**• late infection**			120 (d7)	101 (d 6)					
**pTT (s) ^e^** **Normal: 45** **–** **50 s**									• 47 (d 5 1st pt)
									• Normal (d 7–8 2nd pt) despite heparin
**Thrombin Time (s)** **Normal: 13** **–** **18 s**						Normal throughout			
**Factor II (thrombin) %**						55 (d 10)			
**Prothrombin Time (s)** **Normal: 10** **–** **14 s**				58 (d 6)					
**Prothrombin Index, % **									
**• late infection**			51 (d 7)						
**INR ^f^** **Normal: 0.9** **–** **1.2 [65]**							Normal at admission		
**Plasma fibrinogen (g/L) ^g^** **Normal: 2-4 g/L**									
**• nadir **						0.8 (d 10)			
**• late infection**			0.38 (d 7)	0.4 (d 6)		2.6 (d 13)			
**FDP (mg/L) ^h,i^** **Normal: <10 mg/L**									
**• hospital admission**							Normal		
**• peak **						>40 (d 7–8)			
**• late infection**			>40 (d7)						
**D-dimers (ng/mL) mean** **ULN: ≤400 ng/mL**									
**• hospital admission **								>50,000 F	
								~20,000 NF	
**• peak **								~180,000 F(d 5)	
								~75,000 NF(d 3-5)	
**• late infection**				21,800(d 6)				~65,000 F(d >8)	
								~35,000 NF(d >8)	
**Thrombocytes Normal:** **130-400 × 10^3^/mm^3^ or** **150-450 × 10^9^/L**									
**• hospital admission **				72 × 10^9^/L		150 × 10^9^/L (d 3)	105 × 10^9^/L (d 5)		162 × 10^3^/mm^3^(d 4 1st pt)
**• nadir **						83 × 10^9^/L (d 5)			
**• late infection**	<10,000 mm^3 ^		90 × 10^9^/L (d 6)			227 × 10^9^/L (d 15)	2 × 10^9^/L(d 19)		150 × 10^3^/mm^3 ^(d 6-7, 1st pt)
									253 × 10^3^/mm^3 ^(d 7, 2nd pt)
									205 × 10^3^/mm^3 ^(d 8, 2ndpt)
**Factor V %**						Normal			
**Factor VII/X %**						40 (d 10)			

^a^: F = Fatal ^b^: NF = Nonfatal ^c^: aPTT = Activated partial thromboplastin time ^d^: Thrombofax (Ortho Diagnostic Systes, Raritan, NJ) ^e^: pTT = partial thromboplastin time ^f^: INR = International normalized ratio ^g^: Thrombowellcotest (Wellcome Research Laboratories, England) ^h^: FDP = Fibrin Degradation Products ^i^: Aserachrom D-Di (Diagnostica Stago, Parsippany, NJ)

Hepatic filovirus infection results in hepatocellular necrosis that leads to mild-to-marked elevations in aspartate aminotransferase, alanine aminotransferase, γ-glutamyltransferase, alkaline phosphatase, and lactate dehydrogenase concentrations, [44,45,51,54] and decreases in plasma protein concentrations [41]. Terminal liver failure likely contributes to the clinically evident coagulopathies, secondary to loss of production of vitamin K-associated coagulation factors. Both MARV and EBOV particles have been identified in hepatocytes during the course of natural infection [44,45,51,52,54]. 

Renal function is reported to progressively decline during the first week of infection with a concomitant decrease in urine output resulting in terminal azotemia and oliguria [45,47,48,51,66]. Excess protein (0.68 g/L) and white blood cells (0–4) have been reported in the cerebrospinal fluid from patients with MARV disease [50].

### 2.3. Histopathology

Filoviruses infect a wide variety of parenchymal tissues, but the most often described histopathologically include the spleen, liver, kidneys, adrenal glands, and brain. Findings from the few autopsy reports available from filovirus-infected patients are incomplete [52,67] but report widespread terminal hemorrhage in the mucous membranes, skin, and parenchymal organs, including the stomach and intestines [37,41], associated with widespread areas of parenchymal coagulative necrosis [37,38,41]. 

In accordance with the lymphadenopathy seen in filovirus-infected patients, randomly distributed, multifocal-to-coalescing necrosis characterizes the lesions seen in lymphoid tissues. However, lymphocytes are not infected by filoviruses, and as a result, direct viral infection is not the cause of the massive lympholysis and lymphoid necrosis seen following infection [49,55]. Rather, the mechanism of lympholysis may be apoptotic or pyroptotic lympholysis. Similar necrosis and congestion with fibrin deposition, as seen in the lymph nodes, has been described in both the red and white pulp of the spleen, often obscuring the normal splenic sinusoidal architecture [52,68]. 

Hepatic lesions are described as affecting all lobes and consist of random single cell to panlobular hepatocellular necrosis with prominent intracytoplasmic viral inclusion bodies (ICIBs) [41,43]. In addition to the viral ICIBs, numerous Councilman bodies have been seen in hepatocytes in areas of hepatocellular coagulative necrosis [52]. Differentiating Councilman bodies from viral ICIBs has been achieved through transmission electron microscopy, confirming the presence of filamentous filovirus particles [43,52] in the space of Disse, and intracytoplasmically within hepatocytes and Kupffer cells [69]. 

Diffuse, severe adrenocortical necrosis with eosinophilic viral ICIBs has been reported in humans infected with MARV [32,68]. It has been speculated that hypotension and shock associated with terminal cases of filovirus infection may be secondary to adrenocortical necrosis. The loss of mineralocorticoids (aldosterone) and corticosteroids (cortisol), crucial for the maintenance of normal electrolyte concentrations and vascular tone, would lead to a fatal Addisonian-like crisis in terminally ill patients [38]. 

Bone marrow aspirates have been performed in only a few MARV infected patients. A plasmacytosis (up to 30%) and marked increase in immature megakaryocytes have been described [56] that parallels the increase in atypical lymphocytes in the peripheral blood [70]. However, details describing how this quantitative measure was acquired are not documented, and the assessment of plasmacytosis is assumed to be subjective or semiquantitative [56]. 

Histopathologic examination of the brain has varied from the findings of no significant histopathologic lesions to vascular congestion and hemorrhage to lesions consistent with viral encephalitis (e.g., lymphocytic perivascular infiltrates with gliosis and wide-spread microglial nodules). In most cases, histopathologic findings were not correlated with patients’ clinical neurologic exams [41,52,71].

## 3. Ebola Virus Infection in Baboons and Contrasted with Man and Other NHP Species

### 3.1. Clinical Signs

Baboons experimentally infected with EBOV exhibit both a clinical course and clinical signs that closely resemble that seen in infected humans. An average 6-to 10-day course begins with the sudden onset of fever (40.5–41.8 °C), anorexia, and depression, and progresses to widespread bleeding including hematemesis, epistaxis, and melena. A cutaneous rash, similar to that seen in humans, which starts focally and progresses to a generalized distribution, begins on day 7 in baboons (*Papio hamadryas*) [23,25,26,72] but begins earlier in macaques (*Macaca fascicularis* and *Macaca mulattta*) at 5 days post-inoculation with MARV and EBOV. Interestingly, a cutaneous rash is not typically seen following filovirus inoculation of African green monkeys (*Chlorocebus aethiops subspp.*) [26,73].

As described in humans, a peripheral lymphadenopathy is seen 3–4 days post-inoculation involving the axillary, inguinal, and submandibular lymph nodes both in baboons and macaques, [23,73] and a similar hepatosplenomegaly has also been observed [23,24].

### 3.2. Clinical Pathology

As has been reported in most human EBOV infections, baboons infected with unadapted EBOV develop a neutrophilic leukocytosis with left shift and terminal leukopenia [23]. However, baboons infected with guinea pig-adapted EBOV developed a terminal leukocytosis with blast-like lymphocytes composing 70% of the lymphocyte count rather than a terminal leukopenia. Investigators do not know if these differences are the result of inoculation with a guinea pig-adapted EBOVstrain [25] rather than a naturally occurring EBOV strain. Similar atypical blast-like or immature and plasmacytoid lymphocytes have been observed in the peripheral blood in human filovirus infections [41,55]. In this same baboon study, circulating neutrophils were vacuolated. Cytoplasmic vacuolation of neutrophils is highly suggestive of a toxic change associated with secondary bacterial infection, but no blood cultures or necropsies were performed during the course of the study. As a result, the presence of secondary bacterial infections could not be determined in these baboons [25]. Filovirus-infected macaques also develop a neutrophilic leukocytosis early in infection that progresses to a left shift with a concomitant lymphopenia and monocytopenia during the disease course, as is seen in baboons infected with unadapted EBOV strains and in most human cases [72,73,74]. 

The thrombocyte response in filovirus infection is dependent on the stage of infection and virulence of the virus strain administered. Thrombocytopenia that develops over the course of filovirus infection may resolve, persist, or rebound (thrombocytosis) [72,73,74,75]. A persistent thrombocytopenia has been reported in baboons infected with EBOV with the appearance of immature or giant platelets during the terminal stages (Table 2) [23,25]. Thrombocytopenia and giant platelets are also observed in macaques and African green monkeys infected with EBOV, SUDV, and RESTV [58,72,75]. Interestingly, platelet numbers decreased, but remained in normal range, in macaques infected with MARV [73,74]. 

Only a few investigators have measured coagulation factors in baboons infected with filoviruses, but the methodology utilized is currently out-of-date. Coagulation abnormalities seen in filovirus infected baboons include elevations of thrombin index, prothrombin index, fibrinogen, and fibrin degradation products, consistent with disseminated intravascular coagulation (Table 2) [25,26,73,74,76], and resemble the coagulation abnormalities seen in humans. Widespread hepatocellular necrosis occurs, as in seen in humans, with similar elevations in hepatic enzymes (e.g., aspartate and alanine aminotransferases) [23,25,26,73,74].

**Table 2:** Coagulation Parameters in Baboons Infected with Ebola virus.

Virus, Strain	Ebola, monkey adapted [23,26]	Ebola, guinea pig adapted [25]
Route: Challenge dose	SQ: 20–50 LD_50_	SQ: 100 PFU
# Baboons infected, gender	13 (gender NA)	2 F, 1M
Outcome	Probably died d 9	1 died d 10
		2 died d 11
Thrombin Index, % of baseline		
Normal: 80–120%		
• peak	148% (d 4)	<140% (d 1)
• terminal phase	20% (d 9)	~40% (d 11)
Prothrombin Index, % of baseline	156%	
Normal: 80–120%		
• peak	156.6% (d 4)	>120% (d 3)
• terminal phase	32.3% (d 9)	>20% (d 11)
Fibrinogen (g/L)		
Normal: ~2.5–4.5 g/L		
• peak	5.3 (d 1)	Normal
• terminal phase	1.8 (d 9)	<2 (d 10)
Fibrin Degradation Products (arbitrary units)		
• baseline	0.2	
• peak	3 ( d 8)	
• terminal phase	1.5 (d 9)	
Thrombocytes (10^9^/L)		100 (d 11)
Normal: 200–300 × 10^9^/L		

Renal failure, with concomitant increases in creatinine and blood urea nitrogen concentrations, have been reported in baboons infected with EBOV [25], as has been reported in humans [45,47,48,51,66]. A doubling of blood urea nitrogen and creatinine concentrations to 3 times above the normal range, consistent with terminal renal failure, has been also been described in EBOV-infected macaques late in the disease course [35,72]. 

### 3.3. Histopathology

It is reported that frank hemorrhage is a feature most pronounced in EBOV-infected baboons, while fibrin deposition is the most prominent histopathologic feature seen in EBOV-infected African green monkeys [26,35]. However, the literature to support this statement is lacking. Currently, no published reports of the histopathologic changes in parenchymal organs are available beyond brief descriptions and a single light microscopic image of a lymph node from an EBOV infected baboon. This photomicrograph demonstrates diffuse lympholysis or lymphocyte necrosis, loss of the normal cortical architecture, and mild, draining hemorrhage in the subcapsular and medullary sinuses without prominent fibrin deposition [77]. 

In macaques, the highest titers of MARV are found in the liver, spleen, adrenal glands, and lymph nodes, and these titers have direct correlation with the degree of tissue destruction [73]. By day 5 post-EBOV inoculation in macaques, dense fibrin deposits can be found in the spleen and within the parenchyma and vessels of most tissues including the sinusoids of the liver [57], as is seen in human EBOV infections. Eosinophilic ICIBs are present in the hepatocytes of filovirus infected macaques [73]. In African green monkeys, replicating EBOVs have been demonstrated in the liver and adrenal glands 3 to 4 days post-inoculation [26]. 

Additional EBOV studies that include complete coagulation panels and pathologic findings at the light and electron microscopic level from all major organ systems will be needed to confirm the differences reported in coagulation parameters and fibrin deposition in baboons versus other, more commonly utilized, NHP models of filovirus infection. 

## 4. Conclusions

Currently there are no licensed products for filovirus prophylaxis or treatment. Under the U.S. Food and Drug Administration 2002 “Animal Efficacy Rule” (FDA 21 CFR 601.90), data collected from NHP studies may be used for licensure of therapeutics, provided that the animal model faithfully recapitulates the pathogenesis of the human disease condition [73]. The ultimate goal of these animal model studies is the development of effective countermeasures against these viruses. While the baboon has not been utilized as frequently as the macaque model to study the pathophysiology of filovirus infection in the United States, the baboon model may have certain advantages over these other NHP models and warrants further study. The immunoglobulin profile of baboons more closely models that of humans, very likely making baboons a better model for vaccine efficacy studies than other NHPs. These species-specific differences must be taken into account when choosing a NHP model to recapitulate human filovirus infection, as the animal model chosen can either facilitate or hinder our understanding of the pathophysiology of infection and the development of effective therapeutics. The greatest obstacle for the use of the baboon model of filovirus infection is the possible shortage of available baboons in the United States. 
